# Spatial attention in natural tasks [version 1; peer review: 2 approved with reservations]

**DOI:** 10.12688/molpsychol.17488.1

**Published:** 2022-12-22

**Authors:** Melville Wohlgemuth, Angeles Salles, Cynthia Moss

**Affiliations:** 1 Department of Neuroscience, University of Arizona, Tucson, Arizona, USA; 2 Department of Biological Sciences, University of Illinois at Chicago, Chicago, IL, 60607, USA; 3 Department of Psychological & Brain Sciences, Johns Hopkins University, Baltimore, MD, 21218, USA; 4 Department of Neuroscience, Johns Hopkins University, Baltimore, MD, 21218, USA; 5 Department of Mechanical Engineering, Johns Hopkins University, Baltimore, MD, 21218, USA

**Keywords:** bats, neuroethology, echolocation, chiroptera, superior colliculus

## Abstract

Little is known about fine scale neural dynamics that accompany rapid shifts in spatial attention in freely behaving animals, primarily because reliable indicators of attention are lacking in standard model organisms engaged in natural tasks. The echolocating bat can serve to bridge this gap, as it exhibits robust dynamic behavioral indicators of overt spatial attention as it explores its environment. In particular, the bat actively shifts the aim of its sonar beam to inspect objects in different directions, akin to eye movements and foveation in humans and other visually dominant animals. Further, the bat adjusts the temporal features of sonar calls to attend to objects at different distances, yielding a metric of acoustic gaze along the range axis. Thus, an echolocating bat’s call features not only convey the information it uses to probe its surroundings, but also provide fine scale metrics of auditory spatial attention in 3D natural tasks. These explicit metrics of overt spatial attention can be leveraged to uncover general principles of neural coding in the mammalian brain.

## Introduction

The auditory world of humans and other animals is noisy, complex, and dynamic. From a barrage of acoustic stimuli, an organism must detect, sort, group and track biologically relevant signals to communicate with conspecifics, seek food, engage in courtship, avoid predators, and navigate in space (Bee & Micheyl, 2008; Bradbury & Verenkamp, 2011; Brumm & Slabbekoorn, 2005; Corcoran & Moss, 2017). The success of these natural survival behaviors depends on an animal’s selective attention to stimuli across contexts. How can we monitor rapid shifts in auditory attention of animals engaged in natural tasks? Animals such as echolocating bats that produce and modulate stimulus energy to probe their environments offer powerful solutions to this central challenge in systems neuroscience, because active control over sensing signal features yields quantifiable metrics of overt attention.

Echolocating bats exploit active sensing to localize objects in darkness. They produce sonar signals and process auditory information carried by returning echoes to guide behavioral decisions for a wide range of survival behaviors (Griffin, 1958). There are over 1000 species of bats that use echolocation to forage, find roosts and avoid obstacles, and they occupy all regions of the Earth, outside of arctic zones, from tropical rain forests to savannahs, and from mountains to deserts. Echolocating bat species also show great diversity in diets, from insects to fruit, nectar, blood, and small vertebrates (Denzinger & Schnitzler, 2013; Fenton & Rydell, 2022; Fenton & Simmons, 2015; Kunz & Racey, 1998). Here we focus our discussion on sonar-guided attention in insectivorous bats, which use two main categories of ultrasonic call types, constant frequency (CF) and frequency modulated (FM) (Busnel & Fish, 1980; Fay & Popper, 1995; Griffin, 1958; Schnitzler & Kalko, 2001; Thomas *et al.*, 2004).

Bats compute the direction of objects from differences in echo intensity, spectrum, and timing at the two ears; they compute an object’s distance from the time delay between sonar emission and echo return (Moss & Schnitzler, 1995; Simmons, 1973; Simmons, 1979). Together, this acoustic information gives rise to a 3D representation of the world through sound (Wohlgemuth *et al.*, 2016). Further, the bat makes active adjustments to its echolocation calls in response to 3D spatial information computed from echo returns (Moss *et al.*, 2011), and therefore, the adaptive features of the bat’s calls provide a window to the animal’s attention to objects in the environment.

### Active sensing signals yield a quantifiable metric of attention

Active sensing falls into two broad categories: alloactive sensing, which invokes movement of sensors (eyes, ears, whiskers, among others) to explore sensory stimuli, and homeoactive sensing, which relies on the generation of stimulus energy (sound, electricity) to probe the environment (Zweifel & Hartmann, 2020). Here, we highlight the features of echolocation, a homeoactive sensing system, that offers a quantitative metric of a bat’s moment-to-moment attention to objects in its surroundings. The examples below come from insectivorous bats that produce FM and CF echolocation signals.

### Attention to objects along the horizon

The insectivorous bat’s echolocation calls are directional, forming a spatial beam pattern, emitted through the mouth or nostrils (Ghose & Moss, 2003; Hartley & Suthers, 1987; Hartley & Suthers, 1989; Jakobsen *et al.*, 2012; Jakobsen *et al.*, 2018; Lee *et al.*, 2017). As such, the bat’s sonar beam pattern operates as an auditory flashlight to detect and localize objects in its surroundings. For example, big brown bats (*Eptesicus fuscus*) aim the sonar beam at selected objects with an accuracy of 3–5 deg, maximizing echo returns from prey and simplifying sensorimotor transformations for target interception (Ghose & Moss, 2003). When the bat encounters multiple objects, it shows rapid shifts in the direction of acoustic gaze to steer around obstacles and intercept prey (Surlykke *et al.*, 2009). The bat’s head aim leads its body in flight maneuvers, and it anticipates a target’s future position in a target tracking task, revealing its attention to an object’s trajectory for interception planning (Ghose & Moss, 2006; Salles *et al.*, 2020; Salles *et al.*, 2021). Related work shows that Japanese house bats foraging in the field alternate the sonar beam aim between the direction of flight and the anticipated direction of the next prey interception, suggesting that bats shift biosonar attention between objects by alternating acoustic gaze (Fujioka *et al.*, 2014).

Sonar adjustments in call frequency also reveal attention to objects in the bat’s surroundings. For example, great round-leaf bats, species that use CF-FM echolocation signals, adjust the frequency of their sonar calls to counter the Doppler effect when they fly. These adjustments ensure that echoes return at the frequency to which the emitter is maximally sensitive and is commonly known as Doppler Shift Compensation (Schnitzler, 1968; Schnitzler & Denzinger, 2011). Interestingly, great round leaf bats not only exhibit Doppler Shift Compensation for targets straight ahead, but also for off-axis objects, indicative of spatial attention to obstacles as well as prey (Hiryu *et al.*, 2005).

### Attention to objects along the distance axis

Echolocating bats reduce sonar call duration and the interval between successive emissions as the distance to an object decreases (Griffin, 1958; Griffin *et al.*, 1960; Moss & Surlykke, 2010; Simmons *et al.*, 1979). What function do these call adjustments serve, and how can they be used to infer the bat’s attention to objects along the distance axis? Sound travels in air at a speed of approximately 344 m/s in air, and this results in an echo delay time of approximately 6 milliseconds for each meter of target distance. Bats typically wait for echoes from a selected target to arrive before producing the next call, and the wait time decreases as bats get closer to objects. Thus, the decreasing interval between calls provides an indicator of the bat’s attention to objects along the range axis (Surlykke *et al.*, 2009). However, sonar call interval alone cannot be used to estimate spatial attention to objects, because bats typically wait many milliseconds beyond the arrival of a target echo to produce the next call.

**Figure 1.**
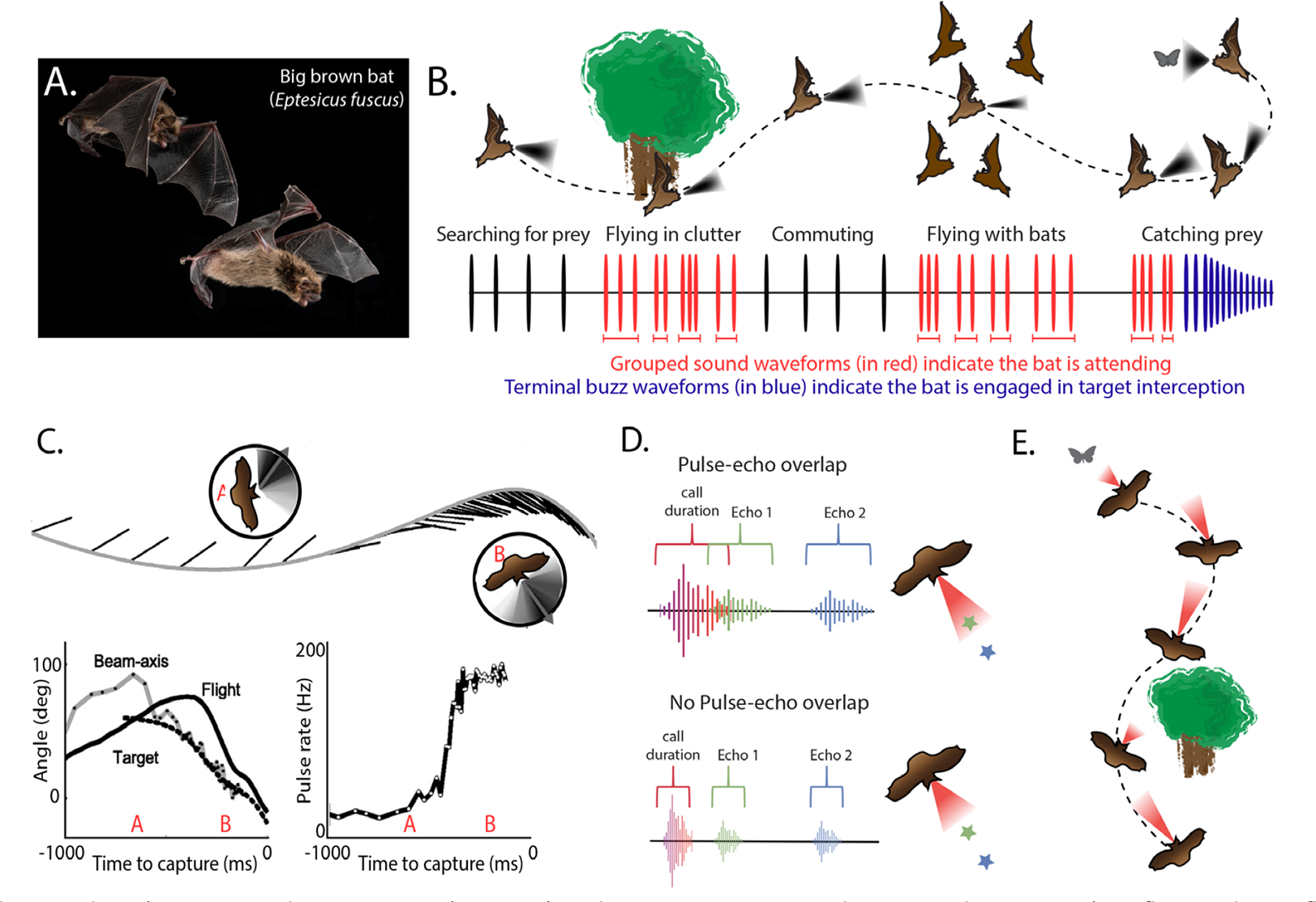
**A**. Photo taken by Dr. Brock Fenton and reused with permission. **B**. Schematic showing a bat flying alone, flying in clutter, commuting, flying with other bats, and chasing prey. The shaded sonar beam pattern illustrates the directional aim of the bat’s sound as it inspects objects. The temporal patterning of calls in each scenario is illustrated in the oscillograms below. Bats searching for prey in open space and commuting produce isolated calls at rate of 5–10/second, and they produce calls at high rates, up to 150–200 sounds/s, during the terminal buzz that precedes prey capture (shown in blue). Bats flying in clutter, with other bats or chasing evasive prey produce clusters of calls, termed sonar sound groups, at rates of 20–80 sounds/s (in red), which index sonar-guided attention to objects. **C**. The aim of the bat’s sonar beam anticipates the direction of flight (Ghose & Moss, 2006), and once a bat selects its prey, it locks its sonar beam to track it with an accuracy of 3 deg (Ghose & Moss, 2003). **D**. Bats also adjust call duration to avoid overlap between calls and echoes, and therefore provide a metric of attention to objects along the range axis. **E**. schematic of call duration adjustments while in flight and attending to clutter or target prey.

A robust indicator of the bat’s attention along the range axis appears in its adjustment to the duration of sonar calls with changing target distance. FM bats actively avoid overlap between their calls and sonar returns to preserve information about targets carried by the features of echoes (Kalko & Schnitzler, 1993; Surlykke *et al.*, 2009). Evidence that FM bats shift their gaze along the range axis by adjusting the duration of calls comes from a laboratory study in which bats performed a dual task, obstacle avoidance and insect capture (Surlykke *et al.*, 2009). The bat received a food reward for finding its way through the opening of a net to access a compartment containing a tethered insect. As the bat approached the net opening, it shortened the duration of its calls to avoid overlap between its calls and net echoes. Once the bat planned its path around the obstacle, but before navigating through the net opening, it increased the duration of its calls as it attended to the more distant prey item. Thus, the bat tolerated overlap of its calls and net echoes as it shifted its acoustic gaze to the food reward behind the net. These active adjustments in call duration provide a direct metric of the bat’s shift in attention to objects along the range axis (Figure 1).

### Tasks that evoke overt sonar-guided attention

As bats approach targets and steer around obstacles, they do not always continuously shorten the interval between successive echolocation calls with decreasing target distance. Instead, when bats perform challenging behavioral tasks, they reliably produce clusters of echolocation calls, flanked by sonar sounds produced at longer intervals (Kothari *et al.*, 2014; Moss *et al.*, 2006; Petrites *et al.*, 2009; Sändig *et al.*, 2014). This distinct temporal patterning of sonar calls has been termed, “sonar sound groups” or “sonar strobe groups,” which are characterized by a series of echolocation calls produced at relatively stable and shorter intervals than the surrounding calls. It is worth emphasizing that the intervals between calls within a sonar sound group depend on the bat’s distance to objects, and therefore the temporal patterning of calls, not absolute call interval, defines sonar sound groups. Because sonar sound groups have been reported in both field and laboratory studies, and the prevalence of sonar call clustering varies reliably with task difficulty, it has been posited that they serve as an indicator of an echolocating bat’s attention to objects. Indeed, sonar sound groups are produced by bats intercepting targets in clutter (Moss *et al.*, 2006), discriminating target texture (Falk *et al.*, 2011), and avoiding obstacles Petrites *et al.*, 2009; Sändig *et al.*, 2014), all behaviors that evoke sonar-guided attention. These observations motivate the hypothesis that sonar sound group production evokes a sharpened 3D representation of sonar objects.

### Attention-driven changes in neural responses

**Figure 2.**
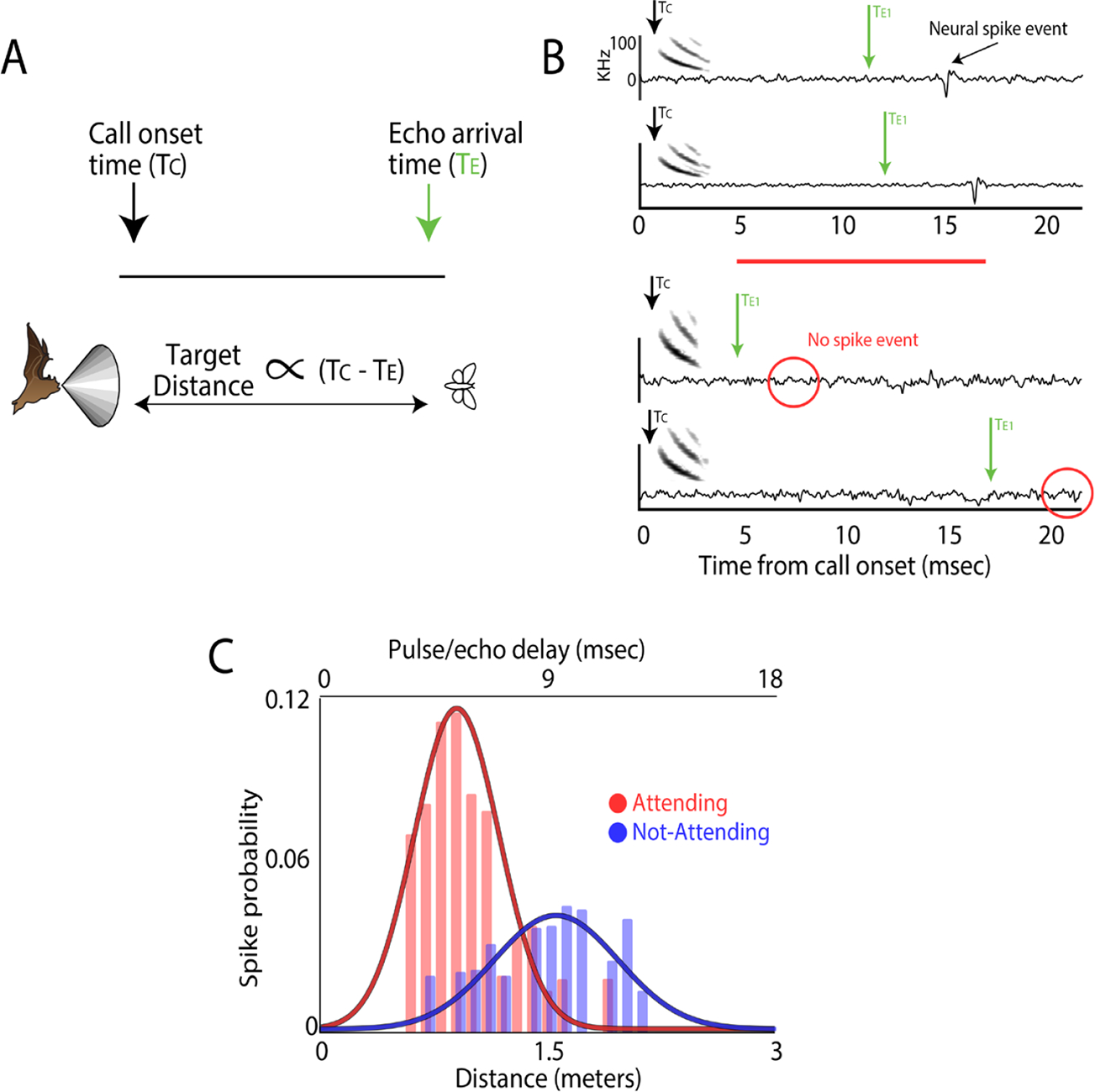
**A**. Schematic depicting target range as calculated from time difference between T_c_ call onset and T_e_ echo arrival. **B**. Example of echo evoked 3D responses of SC neurons and their sharpening with sonar guided attention. Neural spike events occur for specific neurons when the target is at a specific range (delayed tuned neurons, top two panels) but not at other delays (lower two panels, first echo returning at a short delay, where the target is too close to evoke a response from this neuron, and second echo returning at a long delay, where the target is too far away to evoke a response from this neuron). **C**. spike probability for a single neuron. For this neuron the preferred echo delay is ~9 ms (1.5 m range) when the bat is not attending to the target but the spike probability increases, sharpens and is shifted to a shorter delay, ~6 ms (1 m), when the bat is attending.

The hypothesis that sonar sound groups evoke a sharpened spatial representation leads to the prediction that distance coding of sonar objects by single neurons depends on the bat’s sonar call production pattern and corresponding attentional state. Kothari *et al.* (2018) experimentally tested this hypothesis by recording from neurons in the midbrain superior colliculus (SC) of free-flying big brown bats that actively inspected their environments through echolocation. The mammalian SC, homologous to the optic tectum (OT) in other vertebrates, is implicated in species-specific sensorimotor behaviors and spatial attention (Goldberg & Wurtz, 1972; Krauzlis *et al.*, 2013; McIlwain, 1991; McPeek & Keller, 2004; Mysore *et al.*, 2011; Sparks, 1986). In bats, the SC shows specializations that support 3D auditory space representation and acoustic orientation by sonar: A class of neurons in the bat SC responds selectively to the azimuth, elevation and pulse-echo delay, encoding the direction and distance of sonar targets (Valentine & Moss, 1997; Wohlgemuth & Moss, 2016); microstimulation of the bat SC elicits head/pinna movements and the production of echolocation calls (Valentine *et al.*, 2002). Kothari *et al.* (2018) combined neural telemetry, microphone array and high-speed 3D video recordings to study single neuron responses to the azimuth, elevation, and arrival time of echoes from physical objects at the ears of the bat. They used these data to reconstruct 3D spatial response profiles of auditory neurons to echoes arriving from objects as the bat approached and flew around them. Because the bat reveals its attention to objects through active adjustments in its echolocation behavior, neural responses to echoes were sorted with respect to the animal’s sonar-guided attention, indexed by the production of sonar sound groups. This study demonstrated that echoes returning from sonar sound groups (bat attending) evoked sharper echo delay/range tuning to objects than echoes returning from single calls (bat on ‘autopilot’). Importantly, analyses showed that the bat’s attention-driven temporal patterning of calls, not absolute call interval, influenced neural spatial response profiles. These findings support the hypothesis that sonar sound groups evoke a sharpened representation of object distance and feature the dynamics of spatial coding that depend on an animal’s attention to objects in its surroundings (Figure 2).

## Outlook

The echolocating bat’s active adjustments in sonar call features provide a metric to quantify moment-to-moment overt spatial attention to objects and reveals attention-modulated neural coding dynamics. These discoveries open the door to a wide range of comparative studies of spatial cognition and neural representation in animals performing natural tasks.

## Data Availability

No data are associated with this article.
